# Bilateral Tensor Fasciae Suralis Muscles in a Cadaver with Unilateral Accessory Flexor Digitorum Longus Muscle

**DOI:** 10.1155/2017/1864272

**Published:** 2017-01-22

**Authors:** Logan S. W. Bale, Sean O. Herrin

**Affiliations:** Department of Basic Sciences, University of Western States, Portland, OR 97230, USA

## Abstract

Muscle variants are routinely encountered in the dissection laboratory and in clinical practice and therefore anatomists and clinicians need to be aware of their existence. Here we describe two different accessory muscles identified while performing educational dissection of a 51-year-old male cadaver. Tensor fasciae suralis, a rare muscle variant, was identified bilaterally and accessory flexor digitorum longus, a more common muscle variant, was present unilaterally. Tensor fasciae suralis and accessory flexor digitorum longus are clinically relevant muscle variants. To our knowledge, the coexistence of tensor fasciae suralis and accessory flexor digitorum longus in the same individual has not been reported in either cadaveric or imaging studies.

## 1. Introduction

Tensor fasciae suralis (TFS; ischioaponeuroticus) is an accessory muscle of the posterior thigh and popliteal fossa. This muscle is considered rare and its prevalence in the general population is unknown [1]. Several case reports describing TFS have been published previously, including both cadaveric [2–10] and radiological studies [1, 11–13].

Accessory flexor digitorum longus (AFDL) is a muscle variant of the ankle that is more common than TFS. From clinical and cadaveric studies, the prevalence of AFDL has been estimated to be ~6% [14, 15] to 13-14% [16, 17]. This accessory muscle may exist bilaterally; however a unilateral presentation of AFDL is more common [18].

Here we report the bilateral presence of TFS muscles discovered during routine cadaveric dissection as part of chiropractic education. Further investigation revealed a unilateral AFDL muscle. To our knowledge, this is the first published case report of an individual with both TFS and AFDL.

## 2. Case Presentation

**Figure 1:**
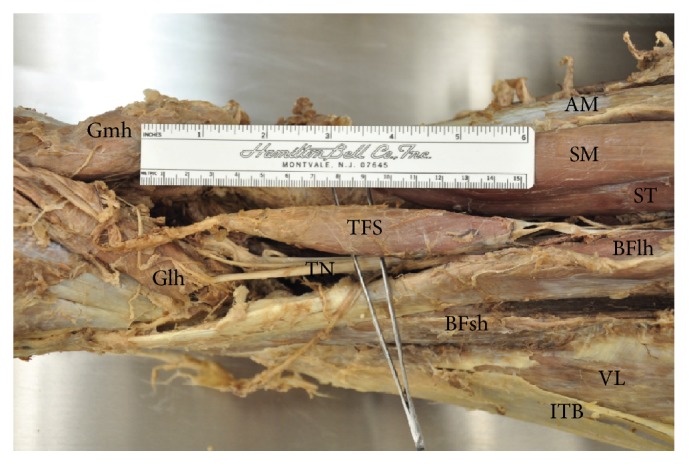
Dissection of right posterior thigh and popliteal fossa to show tensor fasciae suralis muscle (TFS: tensor fasciae suralis muscle; TN: tibial nerve; Gmh: gastrocnemius muscle, medial head; Glh: gastrocnemius muscle, lateral head; AM: adductor magnus muscle; SM: semimembranosus muscle; ST: semitendinosus muscle; BFlh: biceps femoris muscle, long head; BFsh: biceps femoris muscle, short head; VL: vastus lateralis muscle; ITB: iliotibial band).

During educational dissection of a Caucasian, 51-year-old male cadaver, bilateral TFS muscles were identified in the posterior thighs and popliteal fossae. This accessory muscle most commonly arises from the semitendinosus muscle, but TFS can take origin from any of the hamstring muscles. On both sides of the cadaver, TFS arose from the long head of the biceps femoris muscle with a fascial origin measuring approximately 3.3 cm in length (Figure 1). The fusiform belly of each TFS muscle measured approximately 12.0 × 2.5 × 1.0 cm in greatest dimensions. The muscle belly was continuous inferiorly with a fascial component approximately 13.0 cm long that inserted onto the fascia of both heads of the gastrocnemius muscle.

**Figure 2:**
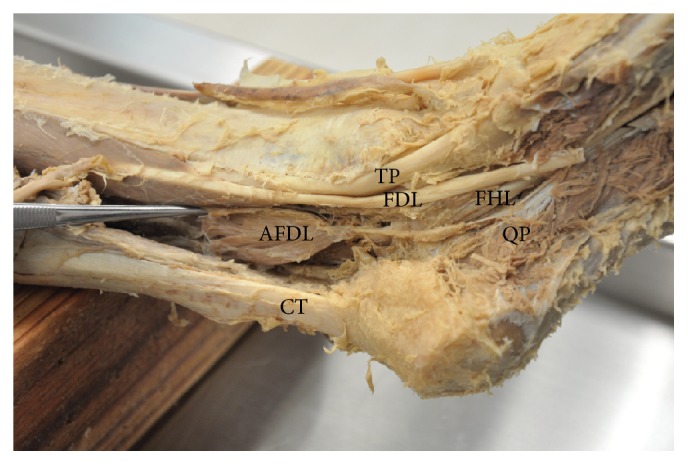
Dissection of left leg and foot to show accessory flexor digitorum longus muscle (AFDL: accessory flexor digitorum longus muscle; CT: calcaneal tendon; FDL: flexor digitorum longus tendon; FHL: flexor hallucis longus tendon; TP: tibialis posterior tendon; QP: quadratus plantae muscle).

Dissection of the left leg and foot revealed the presence of an AFDL muscle, with a bipennate muscle belly that measured 5.2 × 2.3 × 0.3 cm in greatest dimensions (Figure 2). The origin was the deep transverse fascia of the leg and the muscle belly was located posterolateral to the posterior tibial neurovascular bundle. The tendon of insertion was 5.5 cm long and coursed through the tarsal tunnel before blending extensively with the fibers of the quadratus plantae muscle. At the level of the flexor retinaculum the inserting tendon of AFDL was located immediately lateral to the posterior tibial neurovascular bundle.

## 3. Discussion

The muscle variants TFS and AFDL are clinically relevant accessory muscles. The presence of TFS may cause swelling in the popliteal fossa [12] or it may be discovered as an incidental finding during imaging [11]. When TFS presents as a popliteal mass it may mimic a Baker's cyst on physical examination [11]. Due to the muscle's location in relation to the neurovascular elements of the popliteal fossa, the presence of TFS has been speculated to cause compression injuries of the popliteal vein [6] and the sciatic, tibial, and sural nerves [3]. We suspect that the popliteal artery could also be compressed by TFS, as aberrant slips of the gastrocnemius have been shown to cause popliteal artery entrapment syndrome [13, 19–21]. The third head of gastrocnemius (gastrocnemius tertius), a muscle variant more common than TFS, has been reported to cause entrapment of popliteal neurovascular structures [4, 22–24]. If TFS does indeed contribute to entrapment/compressive injuries in the popliteal fossa, resection of TFS may be an appropriate intervention as good surgical outcomes for similar symptoms have been achieved by resection of the third head of gastrocnemius and/or aberrant slips of muscle and tendon in the popliteal fossa [23–25].

Accessory flexor digitorum longus has been reported to be found more commonly in males than females [14]. In some instances, AFDL may be a two-headed muscle [26, 27], although the muscle identified in this case report consisted of only one head. The presence of AFDL is believed to be associated with tarsal tunnel syndrome [15, 26, 28–30], club foot [31], and flexor hallucis syndrome [32]. The fibulocalcaneus (peroneocalcaneus) internus (PCI) muscle of Macalister is a variant muscle that can be easily confused with AFDL. In a case report detailing the presence of a unilateral PCI muscle, Lambert thoroughly described distinguishing differences between the PCI and AFDL muscles, including origin, insertion, course, and position within the tarsal tunnel [33]. Like AFDL and PCI, accessory soleus is a normal muscle variant of the ankle. The accessory soleus muscle usually arises from the soleal line of the tibia [34] and it inserts in one of five common patterns onto the calcaneal tendon or calcaneus [13].

It has been suggested that tensor fasciae suralis arises from muscle primordia that have failed to disappear in the lower limb bud of the developing embryo [9, 10]. Since muscles are inserted more distally in the human embryo compared to their adult form, Kumar postulated that TFS is a muscle that may take the place of embryological fibrous prolongations that exist between the tendon of biceps femoris and the deep fascia of the leg [4]. Conversely, AFDL is speculated to represent an evolutionary remnant of the portion of the flexor hallucis longus muscle that migrated from the leg to the plantar foot to become the medial head of the quadratus plantae muscle [16, 35, 36]. As this case report is the first to describe the synchronous occurrence of TFS and AFDL muscles in the same individual and because of the seemingly separate developmental patterns for these accessory muscles, we do not believe that there is a connection between TFS and AFDL from the point of embryological development. The significance of multiple muscular anomalies in this cadaver is not known; however this case report does exemplify the need for awareness of muscle variants and their relevant clinical implications while performing educational cadaveric dissection, medical imaging, and physical diagnosis.

## References

[1] Kim K. H., Shim J. C., Lee G. J., Lee K. E., Kim H. K., Suh J. H. (2015). MR imaging and ultrasonographic findings of tensor fasciae suralis muscle: a case report. *Journal of the Korean Society of Radiology*.

[2] Barry D., Bothroyd J. (1924). Tensor fasciae suralis. *Journal of Anatomy*.

[3] Gandhi K. R., Wabale R. N., Farooqui M. S. (2015). Bilateral presentation of tensor fascia suralis muscle in a male cadaver. *International Journal of Anatomy and Research*.

[4] Kumar G. R., Bhagwat S. (2006). An anomalous muscle in the region of the popliteal fossa: a case report. *Journal of the Anatomical Society of India*.

[5] Padmalatha K., Prakash B. S., Mamatha Y., Ramesh B. R. (2011). Ischioaponeuroticus/tensor fascia suralis. *International Journal of Anatomical Variations*.

[6] Somayaji S. N., Vincent R., Bairy K. L. (1998). An anomalous muscle in the region of the popliteal fossa: case report. *Journal of Anatomy*.

[7] Tubbs R. S., Salter E. G., Oakes W. J. (2006). Dissection of a rare accessory muscle of the leg: the tensor fasciae suralis muscle. *Clinical Anatomy*.

[8] Turner W. (1884). Absence of extensor carpi ulnaris and presense of an accessory sural muscle. *Journal of Anatomy and Physiology*.

[9] Mudiraj N. R., Dhobale M. R., Joshi U. U. (2012). Tensor fasciae suralis—an unusual variation. *Biomirror*.

[10] Rajendiran R., Murugesan A. (2016). Unilateral tensor fascia suralis: a case report. *Brunei Darussalam Journal of Health*.

[11] Chason D. P., Schultz S. M., Fleckenstein J. L. (1995). Tensor fasciae suralis: depiction on MR images. *American Journal of Roentgenology*.

[12] Montet X., Sandoz A., Mauget D., Martinoli C., Bianchi S. (2002). Sonographic and MRI appearance of tensor fasciae suralis muscle, an uncommon cause of popliteal swelling. *Skeletal Radiology*.

[13] Sookur P. A., Naraghi A. M., Bleakney R. R., Jalan R., Chan O., White L. M. (2008). Accessory muscles: anatomy, symptoms, and radiologic evaluation 1. *Radiographics*.

[14] Cheung Y. Y., Rosenberg Z. S., Colon E., Jahss M. (1999). MR imaging of flexor digitorum accessorius longus. *Skeletal Radiology*.

[15] Sammarco G. J., Stephens M. M. (1990). Tarsal tunnel syndrome caused by the flexor digitorum accessorius longus. A case report. *The Journal of Bone & Joint Surgery—American Volume*.

[16] Nathan H., Gloobe H., Yosipovitch Z. (1975). Flexor digitorum accessorius longus. *Clinical Orthopaedics and Related Research*.

[17] Peterson D. A., Stinson W., Lairmore J. R. (1995). The long accessory flexor muscle: an anatomical study. *Foot & Ankle International*.

[18] Batista J. P., del Vecchio J. J., Golanó P., Vega J. (2015). Flexor digitorum accessorius longus: importance of posterior ankle endoscopy. *Case Reports in Orthopedics*.

[19] Kim H. K., Shin M. J., Kim S. M., Lee S. H., Hong H. J. (2006). Popliteal artery entrapment syndrome: morphological classification utilizing MR imaging. *Skeletal Radiology*.

[20] Liu P. T., Moyer A. C., Huettl E. A., Fowl R. J., Stone W. M. (2005). Popliteal vascular entrapment syndrome caused by a rare anomalous slip of the lateral head of the gastrocnemius muscle. *Skeletal Radiology*.

[21] Molinaro V., Pagliasso E., Varetto G. (2012). Popliteal artery entrapment syndrome in a young girl: case report of a rare finding. *Annals of Vascular Surgery*.

[22] Bergman R. A., Walker C. W., El-Khour G. Y. (1995). The third head of gastrocnemius in CT images. *Annals of Anatomy*.

[23] Connell J. (1978). Popliteal vein entrapment. *British Journal of Surgery*.

[24] Iwai T., Sato S., Yamada T. (1987). Popliteal vein entrapment caused by the third head of the gastrocnemius muscle. *British Journal of Surgery*.

[25] Gourgiotis S., Aggelakas J., Salemis N., Elias C., Georgiou C. (2008). Diagnosis and surgical approach of popliteal artery entrapment syndrome: a retrospective study. *Vascular Health and Risk Management*.

[26] Gümüşalan Y., Kalaycioğlu A. (2000). Bilateral accessory flexor digitorum longus muscle in man. *Annals of Anatomy*.

[27] Holzmann M., Almudallal N., Rohlck K., Singh R., Lee S., Fredieu J. (2009). Identification of a flexor digitorum accessorius longus muscle with unique distal attachments. *Foot*.

[28] Canter D. E., Siesel K. J. (1997). Flexor digitorum accessorius longus muscle: an etiology of tarsal tunnel syndrome?. *Journal of Foot and Ankle Surgery*.

[29] Duran-Stanton A. M., Bui-Mansfield L. T. (2010). Magnetic resonance diagnosis of tarsal tunnel syndrome due to flexor digitorum accessorius longus and peroneocalcaneus internus muscles. *Journal of Computer Assisted Tomography*.

[30] Saar W. E., Bell J. (2011). Accessory flexor digitorum longus presenting as tarsal tunnel syndrome: a case report. *Foot & Ankle Specialist*.

[31] Dobbs M. B., Walton T., Gordon J. E., Schoenecker P. L., Gurnett C. A. (2005). Flexor digitorum accessorius longus muscle is associated with familial idiopathic clubfoot. *Journal of Pediatric Orthopaedics*.

[32] Eberle C. F., Moran B., Gleason T. (2002). The accessory flexor digitorum longus as a cause of Flexor Hallucis Syndrome. *Foot and Ankle International*.

[33] Lambert H. W., Atsas S. (2010). An anterior fibulocalcaneus muscle: an anomalous muscle discovered in the anterior compartment of the leg. *Clinical Anatomy*.

[34] Bergman R. A. (2016). *Bergman's Comprehensive Encyclopedia of Human Anatomic Variation*.

[35] Winckler G., Giacomo G. (1955). La veritable terminaison de la chair carree de Sylvius. *Acta Anatomica*.

[36] Lewis O. J. (1962). The comparative morphology of M. flexor accessorius and the associated long flexor tendons. *Journal of Anatomy*.

